# Real life rates of sustained virological response (SVR) and predictors of relapse following DAA treatment in genotype 3 (GT3) patients with advanced fibrosis/cirrhosis

**DOI:** 10.1371/journal.pone.0200568

**Published:** 2018-07-31

**Authors:** Alessandra Mangia, Ruggero Losappio, Giovanni Cenderello, Domenico Potenza, Michele Mazzola, Giulio De Stefano, Natalia Terreni, Massimiliano Copetti, Nicola Minerva, Valeria Piazzola, Donato Bacca, Vincenzo Palmieri, Fernando Sogari, Rosanna Santoro

**Affiliations:** 1 Liver Unit, “Casa Sollievo della Sofferenza”, IRCCS, San Giovanni Rotondo, Italy; 2 Infectious Disease Unit “Vittorio Emanuele II” Hospital, Bisceglie, Italy; 3 Infectious Disease Division “Galliera” Hospital, Genova, Italy; 4 Infectious Disease Unit “A.Perrino” Hospital, Brindisi, Italy; 5 Infectious Disease Unit “Madonna delle Grazie” Hospital, Matera, Italy; 6 Gastroenterology Unit,” Valduce” Hospital, Como, Italy; 7 Biostatistic Unit,“Casa Sollievo della Sofferenza”, IRCCS, San Giovanni Rotondo, Italy; 8 Internal Medicine Unit “Caduti di Guerra” Hospital, Canosa, Italy; 9 Gastroenterology and Hepatology Outpatients clinic, Casarano, Italy; 10 Internal Medicine “A Murri” Pavillion, University of Bari, Bari, Italy; 11 Internal Medicine “Santissima Annunziata” Hospital, Taranto, Italy; National Taiwan University Hospital, TAIWAN

## Abstract

**Background:**

Treatment of GT3 remains challenging compared to other genotypes.

**Aims:**

To explore real life SVR rates and to identify predictors of virological failure across the most recently used Direct acting antiviral (DAA) regimens in a large cohort of Italian patients with cirrhosis or advanced fibrosis (F3 or F4).

**Methods:**

Between May 2015 and June 2017, the combinations of sofosbuvir (SOF) plus daclatasvir (DCV) ± RBV and SOF plus velpatasvir (VEL) ± RBV become available in our Country. Patients were treated following Italian guidelines within a protocol implemented by 11 centers working together on genetics.

**Results:**

Of 336 patients, 38.1% were Peg/IFN-experienced. SOF/DCV was used in 65.1%, SOF/VEL in the remaining. Overall SVR12 was 90.2% ranging from 87.2% after SOF/DCV to 95.7% after SOF/VEL (p = 0.012). No additional benefits of RBV use were observed for both regimens. 155 patients (46.1%) had cirrhosis. SVR12 was 87.1% (135/155) for cirrhotic patients and 92.8% (169/182) for non-cirrhotic (p = 0.09). NS5A-RASs were present at baseline in 6.4% of patients, PNPLA3GG and IL28BCC genotypes in 7.3% and 33.0%, respectively. No association between favorable genetics and SVR12 was observed. Predictors of relapse were: history of Peg/IFN/RBV failure (OR = 6.34, 95% CI 2.04–19.66, P = .001), baseline NS5A-RASs (OR = 8.7, 95% CI 1.58–47.92, P = 0.013) and treatment regimen (OR = 5.57 95% CI 1.64–18.95.96, P = 0.006).

**Conclusions:**

Our real-world results validate the efficacy of current GT3 IFN-free regimens suggesting that, among patients with severe disease, Peg/IFN/RBV experience and NS5A associated RASs are predictors of relapse. Their relevance can be expected to decline with the use of SOF/VEL. (250).

## Introduction

GT3 represents one of the last challenges in the field of HCV treatment [1]. The association with steatosis, responsible for an accelerated progression to fibrosis, and with rates of HCC higher than in other genotypes [2] justifies the efforts to treat GT3-infected patients as early as possible—these representing 25% of all infections [3]. DCV/SOF [4] and, more recently, SOF/VEL [5,6] led to an increase in the cure rate over the combination of SOF/RBV, yet the proportion of GT3 responding to DAA remains lower than for other genotypes [7]. Therefore, identification of baseline predictors of response is desirable.

IL28BCC and PNPLA3GG genetic polymorphisms are considered predictors either of treatment response or progression of steatosis. In more detail, rs12979860 IL28BCC polymorphism has been associated with both, higher responses to IFN-based therapies and advanced disease in chronic HCV infection [8,9]. PNPLA3 encoding hydrolase against triglycerides and retinyl esters in hepatic stellate cells is associated with genetic variability. The rs738409 PNPLA3GG polymorphism is a risk factor for fatty liver and a marker of steatosis to fibrosis progression for chronic HCV-infected patients [10]. No data on genetics’ impact on GT3 IFN-free treatment are currently available.

Resistance associated substitutions (RASs) are point mutations associated in vitro with DAA resistance. When combined with other negative predictors, RASs may increase the number of failures [11]. Of the different techniques used to detect RASs, population level sequencing has shown clinical relevance [12]. In US, for GT3-infected, naïve and treatment experienced non-cirrhotic patients, baseline testing for NS5A-RAS is recommended to intensify treatment administering RBV in case of RASs [13]. Contrastingly, not to limit access, EASL guidelines do not recommend baseline RASs evaluation, although encouraging it when available, and suggest RBV in cirrhotics and in treatment experienced without cirrhosis [14].

In Italy, costs for 24 weeks of IFN-free treatments were similar to the 12 weeks’ leading to a large use of extended regimens [15]. No other prospective studies on SVR after SOF/DCV for 24 weeks are available.

We analyzed the role of possible predictors of virological failure (including NS5A-RASs, PNPLA3 and IL28B polymorphisms) in a GT3 real life Italian cohort of F3/F4 fibrosis stage patients, treated following Italian guidelines.

## Patients and methods

### Study design

This is an open label prospective observational study on HCV GT3 patients with F3/F4 fibrosis consecutively treated with SOF/DCV or SOF/VEL combinations at 11 centers in Italy (9 from the South and 2 from the North) according to standard clinical practice. Data were prospectively collected in a central electronic database. The first patient started treatment on May 22nd, 2015 and the last on June, 1st 2017. Patients older than 18 years, with documented advanced fibrosis/cirrhosis, including decompensated were eligible. Patients with HIV infection, active HBV, untreated HCC or DAA-experienced were excluded. Naïve and Peg-IFN/RBV-experienced received SOF/DCV for 24 weeks ±weight-based RBV (1000 mg for body weight of up to 75 kg, 1200 for body weight >75 Kg) since DCV availability on May 2015 or SOF/VEL for 12 weeks ±weight-based RBV (1000 mg for body weight of up to 75 kg, 1200 for body weight >75 Kg) since the availability on May 2017. Median Follow-up of patients was 23 months. As this was not a randomized trial, treatment duration and RBV addition were chosen by physicians in accordance with local guidelines and patients’ characteristics. Approval of the study by Ethic Committee was not required as no modifications of local guidelines on management and treatment were expected in the study. Moreover, the data were collected as part of routine clinical care. Any Author had access to patient identifying information during the course of this study. Local data protection committee was consulted prior to analyzing these data. Patients signed an informed consent for genetic testing. Patients’ biochemistry, hematology and virology was assessed at baseline, at week 4 on-treatment and 4 and 12 post-treatment.

### Assessment of liver disease severity

The presence of F3/F4 fibrosis was defined by transient elastography (TE) and/or liver biopsy graded according to Scheuer’s classification system [16]. TE threshold to define cirrhosis or advanced fibrosis were ≥12.5 and 10.1 KPa, respectively.

### Laboratory assessments

Screening assessments included central serum HCVRNA assessment at baseline, IL28B genotyping and PNPLA3 genotyping in addition to standard laboratory clinical tests. HCVRNA levels were measured using RealTime (ART) (Abbott) with 12 IU/ml Low Limit of Quantitation (LLOQ) and 10–12 IU/ml Low Limit od Detection (LLOD). IL28B and PNPLA3 polymorphisms were determined from patients serum at coordinating center by PCR amplification and sequencing. IL28B rs12979860 and PNPL3 rs738409 single-nucleotide polymorphisms were detected as described [17,18]. On-treatment assessments performed at individual center included standard laboratory testing HCVRNA, vital signs and physical examination. The type of previous response to Peg-IFN/RBV was defined by standard criteria [19].

### Sequencing for RASs assessment

Initial genotyping was performed at screening using Innolipa 2.0 assay. Serum samples of all patients at baseline and at relapse were genotyped by Sanger sequencing method. Regions encoding the *NS5*, the *NS5B* and the *NS3* protease gene were amplified, and population-based sequenced. Sequencing and data analysis were performed at coordinating center following the established protocols [18]. As results were not available before the start of treatment, they were not used to guide physician’s decisions. Samples taken 4 weeks after relapse or later were sequenced to exclude re-infection and to identify potential RASs developed at relapse.

### Efficacy endpoints

The primary efficacy endpoint was SVR12, defined as HCVRNA below lower limit of quantification (LLOQ), at post-treatment week 12. Virological failure categories included relapse (HCVRNA > LLOQ at any post-treatment visit in patients with HCVRNA <LLOQ at the end of treatment) and virological breakthrough (HCVRNA ≥ LLOQ on-treatment following HCVRNA < LLOQ).

### Safety assessment

Safety endpoints included graded adverse events (AEs), serious AEs, discontinuations due to AEs, deaths and laboratory abnormalities. The safety population consists of patients who completed antiviral therapy. Data on safety issues were collected during treatment and the 12 weeks of follow up.

Anemia was managed by 200 mg RBV dose reduction. Blood transfusions and erythropoietin were allowed.

### Statistical methods

**Fig 1:**
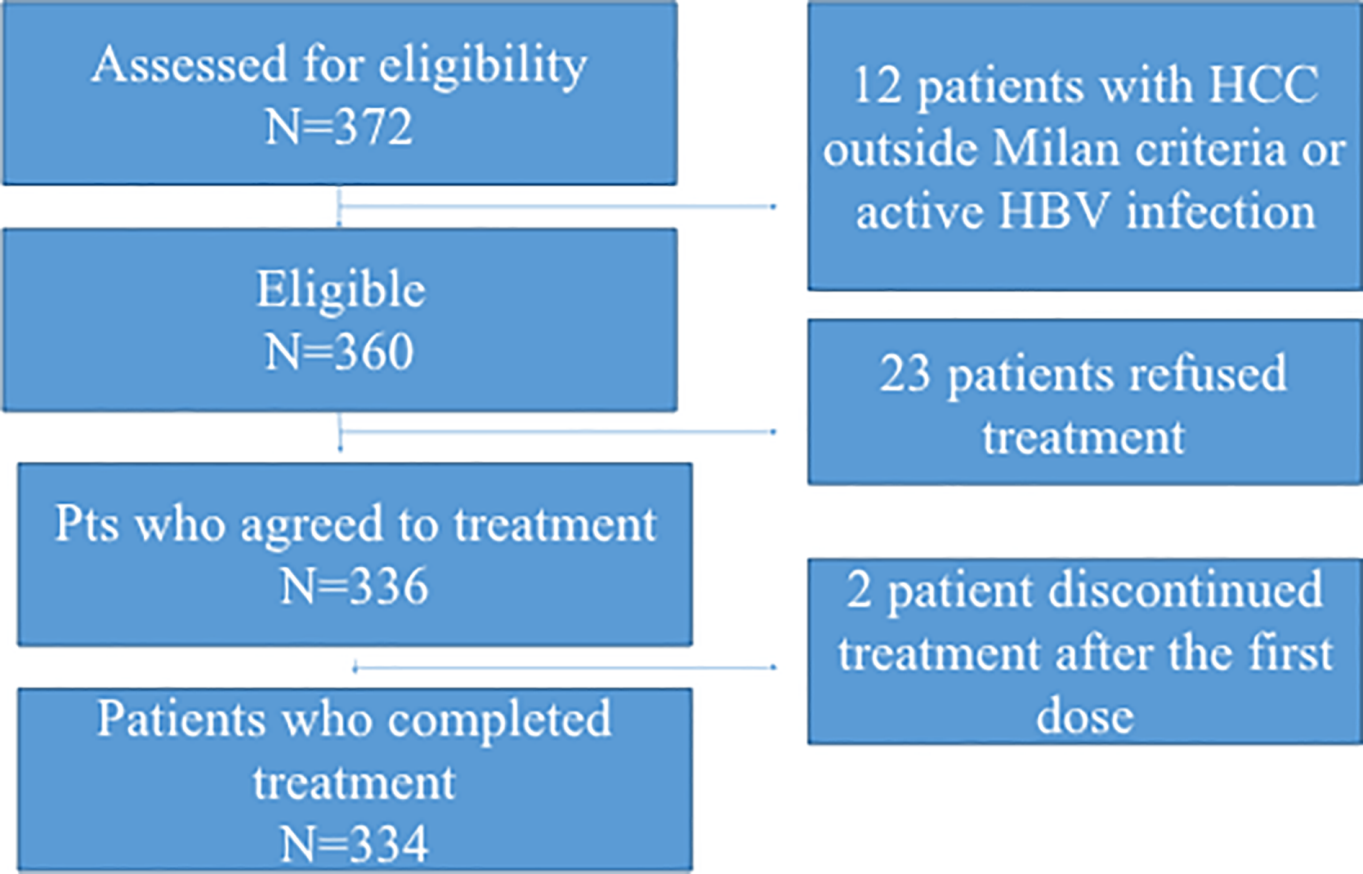
Study flow chart.

Demographic and clinical data were reported as mean and standard deviation on frequencies and percentages for continuous and categorical variables, respectively. Group comparisons were performed using Mann-Whitney U-test and Person chi-square or Fisher exact test for continuous and categorical variables, respectively. Point estimates of rates of SVR12 along their exact 95% Confidence Intervals (CIs) were estimated. SVR were calculated according to the modified intention-to-treat (mITT population). The modified intention-to-treat population included who should have reached a follow up visit at the time of final data analysis including patients who prematurely discontinued treatment with no on treatment virological failure (Fig 1). A multivariate logistic regression model was used to assess lower SVR predictors. Risks were reported as odds ratios (OR) along with 95% confidence intervals (95% CI). A p-value < 0.05 was considered as statistical significant. All analyses were performed using SPSS version 22.0 (IBM corporation, New York, USA).

## Results

### Patients’ characteristics

In total, 372 GT3 patients with F3/F4 fibrosis were assessed for eligibility to treatment at the 11 participating centers. Study chart is shown in Fig 1. Of 360 eligible patients, 24 due to active intravenous drug use, preferred to postpone treatment. Of 336 patients who agreed to treatment, 2 discontinued after the first dose for personal reasons. No patient was lost of follow-up. As shown in Table 1, of 336 analyzed, 219 received SOF/DCV ± RBV and 117 received SOF/VEL ± RBV. In more detail, 60 (27.4%) received RBV with DCV, only 21 (17,9) with VEL. Of 155 (46.1%) participants with cirrhosis, 100 (45.6%) were treated with SOF/DCV and 55 (47.0%) with SOF/VEL.

**Table 1:** Baseline features of patients overall and by treatment regimen.

	Overall N = 336	SOF/SOF N = 219	SOF/VEL N = 117	P value
**Male (n, %)**	274 (81.5)	173 (79.0)	101 (86.3)	0.75
	62 (18.4)	46 (21.0)	16 (13.6)	
**Age (mean, range)**	50.5	50.7	49.2	0.45
	18–87	26–87	18–69	
**BMI** **(mean, range)**	25.8	25.7	26.7	0.89
	18–41	17–41	20–60	
**Cirrhosis**	155 (46.1)	100 (45.6)	55 (47.0)	0.61
**HCV RNA baseline** **(Log 10 mean ± SD)**	5.78 ± 0.88	5.75 ± 0.91	5.72 ± 0.95	0.88
**PLT counts (x10^3^/μl±SD)**	158.9 ± 87.3	160.0 ± 98.9	168.2 ± 72.1	0.29
**CTP class score for cirrhosis** **B7-9**	20 (11.9)	13 (13.0)	3 (10)	0.44
**HCC at baseline**	14 (4.1)	7 (3.2)	3 (4.8)	0.60
**Treatment history**				
**Naive**	208 (61.9)	136 (62.1)	72 (61.5)	0.38
**Relapsers**	47 (14.0)	34 (15.5)	13 (11.1)	
**Non responders**	81 (24.1)	49 (22.4)	32 (27.4)	
NPLA GG*	19/257 (7.3)	10/158 (6.3)	9/109 (8.2)	0.46
IL28BCC*	85/257 (33)	53/158 (33.5)	32/109 (29.3)	1.0
**RBV no**	214 (63.6)	159 (72.9)	96 (82.0)	0.06
**yes**	122 (36.0)	60 (27.3)	21 (17.9)	

*Patatin-like phospholipase domain-containing protein 3 (PNPLA) and Interleukin 28B (IL28B) genotyping were performed in 257 patients

Baseline demographics and treatment characteristics were well balanced across the two treatment groups with the only exception of RBV addition (Table 1). The study population was mostly male (81.5%), with a mean age of 50.5 years (18–87). The proportion of drug users was 34%, of them only 9% were active consumers. Overall, 53.9% of population, including 11.1% with extrahepatic manifestations of HCV, had F3 fibrosis. Of cirrhotic patients, 20 (12.19%) had a Child-Turcotte-Pugh (CTP) score B, the remaining 135 patients A. Overall, 81 of 155 (52.2%) cirrhotics had platelets <100,000/μl and 42 (27.0%) had albumin levels <3.5 g/dl. The most frequent co-morbidity was diabetes, observed in 13.7% of patients.

Patients’ characteristics were comparable across the two regimens. Despite SOF/VEL was available 2 years later than SOF/DCV, rates of cirrhotics treated with SOF/VEL or SOF/DCV were similar.

### Virological response overall and by treatment regimen

The overall SVR12 was 90.2%. Patients’ characteristics by response are reported in Table 2. SVR12 was achieved by 135 of 155 patients with F4 (87.1%). Overall, SVR12 rates were not significantly different in patients with F3 (92.8%, p = 0.09). Naïve patients achieved SVR12 in 95.6% of cases. The corresponding rate for Peg-IFN/RBV-experienced patients was 82.0% (p<0.001).

**Table 2:** Factors associated with sustained virological response (SVR) or virological failure/relapse.

	Non SVR N = 33 (7.2%)	SVR N = 303 (90.2%)	P value
**Male (n, %)**	28 (84.8)	247 (90.5)	1.0
**Age (mean±SD)**	50.6±5.9	50.4±8.5	0.10
**BMI (mean±SD)**	26.6±3.6	25.7±4.1	0.90
**Cirrhosis** **Advanced fibrosis**	20 (60.6)	135 (44.5)	0.098
	13 (39.3)	168 (55.4)	
**HCV RNA pre-tx Log10(mean±SD)**	7.0±0.85	5.7±0.88	0.0001
**PLT counts (x10**^**3**^**/ul)** **(mean±SD)**	150±22.8	161±5.2	0.79
**Albumin (g/dl)**	3.7±0.5	4.4±5.0	0.57
**Treatment regimen**			
**SOF/DAC**	28 (84.8)	191 (63.0)	0.012
**SOF/VEL**	5 (15.2)	112 (37.0)	
**Treatment history**			
**Naïve**	9 (27.3)	194 (64.0)	0.0001
**Prior NR/Relapsers**	24 (72.7)	109 (36.0)	
PNPLA GG*	3 (9.6)	16 (5.2)	0.41
IL28B CC*	8 (25.8)	77 (25.4)	0.83
**NS5A RAS**°	6 (19.5)	12 (4.0)	0.009

*Patatin-like phospholipase domain-containing protein 3 (PNPLA) and Interleukin 28B (IL28B) genotyping were performed in 257 patients

° available in 275 patients

Among cirrhotics, the analysis showed 92.6% SVR for naïve and 78.6% for Peg-IFN/RBV-experienced (p = 0.015). 96.3% of stage 3 naïve achieved SVR12. This rate was higher than in F3 patients Peg-IFN/RBV-experienced (87.5%) (p = 0.037).

Thirty-one patients experienced relapse resulting in virological failure. Two CPT B cirrhotics with ascites and septic complications died 8 and 12 weeks after relapse, respectively, both of them had been treated with SOF/DCV.

**Fig 2:**
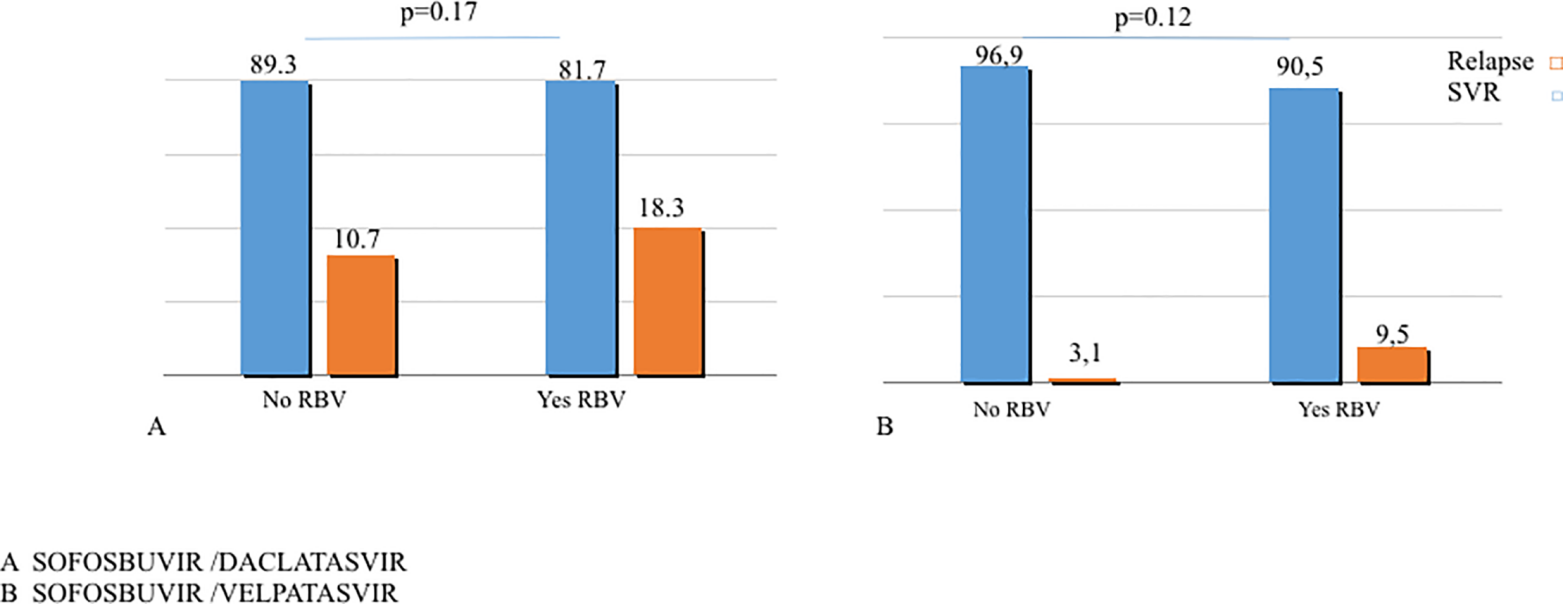
Sustained Virological Response (SVR) by DAA treatment regimen in the subgroup of patients with cirrhosis. The figure shows Sustained Virological Response (SVR) rates in patients with cirrhosis treated with SOF/DCV or SOF/VEL without RBV.

**Fig 3:**
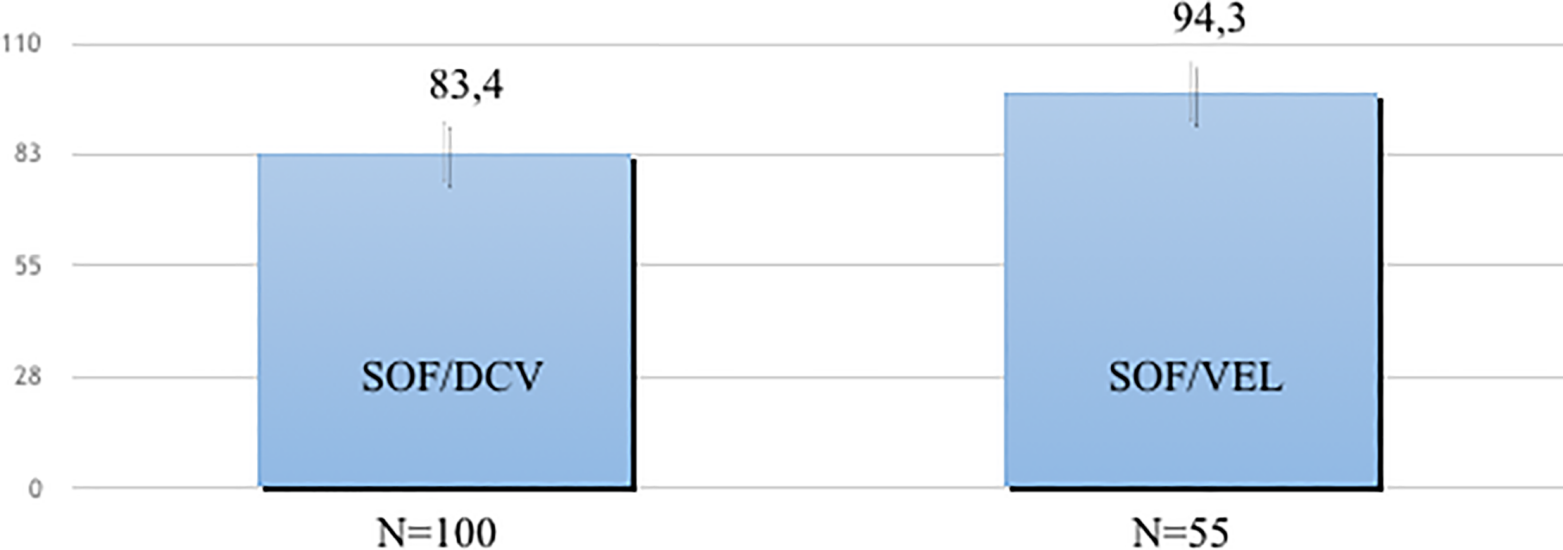
SVR by RBV. The figure shows Sustained Virological Response (SVR) rates with (1) or without (2) RBV in patients treated with SOF/DCV (A) or with SOF/DCV (B).

SVR12 were 95.7% among patients treated with SOF/VEL and 87.2% among patients receiving SOF/DCV (p = 0.012), due to higher SVR12 in the subgroup of F4 treated with SOF/VEL. Subgroup analysis by treatment regimen showed 94.5% SVR12 for F4 patients treated with SOF/VEL and 83.0% for those treated with SOF/DCV (p = 0.046) (Fig 2). The corresponding rates among patients with F3 fibrosis were similar: 96.8% and 90.8% (p = 0.22). Patients treated with SOF/DCV received RBV in 60 cases, 45 of them had cirrhosis. No significant difference in SVR were observed according to RBV use in SVR12 as 87.3% of F4 patients treated for 24 weeks without RBV vs 77.8% of patients treated with RBV achieved SVR12. Fig 3 depicts SVR12 by RBV use and treatment (A = SOF/DCV, B = SOF/VEL). The very small number of patients treated with RBV in the SOF/VEL group suggests that in real life physicians are only partially following guidelines. Only 1 of 16 patients treated with SOF/VEL (mITT) and RBV did not achieve SVR. This rate was similar to 5.1% rate in the corresponding group of F4 patients who did not receive RBV in combination with SOF/VEL (p = 1.0).

Within SOF/DCV treated patients, SVR12 among F3 subjects was 93.4% in naïve and 76.8% in previously treated (p = 0.001); the corresponding rates in SOF/VEL were 97% in naïve and 94.1% in Peg-IFN/RBV failure (p = 0.65).

Across the two regimens SOF/VEL was associated with higher SVR12 in previously treated F4 subjects (91.3% vs 71.1%) (p = 0.09).

In the subgroup of naïve patients treated with SOF/DCV and RBV, SVR12 were 93.6%, as compared to 92.6% without RBV (p = 0.59). Among previously treated, the corresponding rates were 72.6% with and 72.7% without RBV (p = 1.0).

No significant differences in SVR12 were evident across the two treatment regimens for non-cirrhotic naïve (96% vs 97.2% for SOF/DCV and SOF/VEL, respectively, p = 1.0). For F3, Peg-IFN/RBV-experienced SVR12 were numerically higher in SOF/VEL group (96.0% vs 81.8%, p = 0.14). However, due to the small number of patients in this group, difference was not statistically different.

### Sequencing assessments

Of 336 patients, 275 had an available sample for RASs assessment at baseline. All DAA failure patients were re-evaluated for RASs. Sequencing confirmed the virological failures were due to relapse in all but 2 cases. Both of them were active drug users. Overall, 31 patients had NS5A-RASs (9.4%). NS5A-RASs were observed in 21 patients at baseline (6.4%) and post-treatment in additional 10 patients. NS5A position 30 (A30K/S/V) were the most commonly observed variants.

Overall, 16 patients had Y93H variant at baseline alone or in combination with A30V and 7 developed it at the time of relapse. Twelve of 21 (57.1%) patients with baseline RASs achieved SVR, as compared to 84.7% of those without NS5A baseline RASs (p = 0.004.) (Fig 2). 13 of 14 patients with Y93H who were treated with DCV did not achieve SVR (92.9%). Two out of 3 patients with A30K treated with SOF/DCV achieved SVR (66.7%). Two of 2 patients with Y93H who received SOF/VEL (100%) achieved SVR. Subgroup analysis of SOF/VEL, showed baseline A30Kin 5 and Y93H in 2, all achieved SVR12. None of them had received RBV. In 3 of 21 (14.2%) (16%) patients with baseline NS5A-RASs, NS3 RASs were also observed. No NS5B nucleoside inhibitor-induced RASs were observed in any case, at failure (data not shown).

The analysis of RASs by treatment regimen showed that among subjects treated with SOF/DCV, 12 had baseline RASs either A30K or Y93H. SVR was attained in 75% of subjects, all treated for 24 weeks without RBV. In the group of SOF/VEL 100% of 9 patients with A30K or Y93H attained SVR. Up to 92.4% of F3 patients without RASs achieved SVR; the corresponding rate among F3 patients with baseline RASs was 62.5% (p = 0.050). Overall, SVR12 was 75.8% in patients with F4 and baseline RASs and 50% in patients with both these unfavorable features (50%) (p = 0.08).

### Characteristics of patients with virological failure

Of 33 treatment failure, two were patients who discontinued treatment after the first dose of SOF/VEL and were considered non responders. 31 patients (mITT) experienced a relapse, 18 were F4 (58.1%); all but 1 in CTP class A. 28 were treated with SOF/DCV, of which, one was in CTP B class in the waiting list for OLT; the remaining were in CTP class A. Thirteen non cirrhotic patients had relapse, 9 were Peg-IFN/RBV-experienced and 3 had baseline NS5A-RAS; 12 patients (92.3%) failed SOF/DCV, 1 SOF/VEL. Keeping in mind that the different treatment groups are not matched and that this study was not powered to compare efficacy between SOF/DCV and SOF/VEL regimens, results suggest lower relapse rates, despite the presence of compensated cirrhosis, in the SOF/VEL group. 10 (30.3%%) patients with virological failure had baseline NS5A-RASs.

### Univariate analysis of predictors of SVR12

Multiple parameters were tested for possible association with SVR12 in patients with GT3 and advanced liver disease: gender, age, platelet counts, baseline HCVRNA, transaminases values, albumin values, co-morbidities including diabetes, and obesity, fibrosis stage, baseline NS5A-RASs, PNPLA3 and IL28B polymorphisms, treatment history and regimen. Predictors of lower SVR12 are reported in Table 2.

### Multivariate analysis of predictors of SVR12

Multivariate analysis included fibrosis stage, treatment history, treatment regimen, baseline HCVRNA, platelet counts, albumin and NS5A-RAS. Among them, independent predictors of relapse were Peg-IFN/RBV failure (OR = 7.36, 95% CI 2.21–24.48, p = 0.001), baseline NS5A-RASs (OR = 8.40 95% CI 1.51–47.01, p = 0.015), and treatment regimen (OR = 4.49 95% CI 1.30–15.52, p = 0.018) (Table 3).

**Table 3:** Independent predictors of virological failure in GT3 patients treated with IFN-free regimen.

			95% CI	
	Sig	Expo (B)	Lower	Upper
**Cirrhosis**	0.19	2.05	0.69	6.08
**NS5A RASs**	0.013	8.71	1.58	47.92
**Tx history**	0.001	6.34	2.04	19.66
**Tx regimen**	0.006	5.57	1.64	18.95
**PLT count**	0.88	1.00	0.99	1.00
**Age (yrs)**	0.99	1.00	0.94	1.07
**HCV RNA IU/ml**	0.57	1.00	1.00	1.00

### Safety

Overall, 78% of participants experienced AEs: 58% were considered treatment related as they were not reported in the past and disappeared within 4 weeks after the end of treatment. The proportion of patients with AEs was lower among SOF/VEL than in SOF/DCV. The most common AEs, occurring in at least 33.3% of subjects, was fatigue. Anemia, headache, nausea, insomnia, rash were also frequent. As previously described, in patients receiving RBV, the most common serious AE was anemia [7]. RBV dose reduction was required in 12.5% of patients treated with SOF/DCV combination. Anemia was observed in 31 of patients treated with SOF/DCV; all were on RBV. Anemia was moderate in 20 cases and mild in the remaining 11. No discontinuations or blood transfusions were required. Moderate anemia was developed in F4 female at a higher rate than in male (90.9% vs 44.4%, p = 0.03) after SOF/DCV/RBV. Two of 5 patients on SOF/VEL/RBV reported anemia. This rate was not different from the 50.8% (31/61) observed in SOF/DCV group (p = 0.64). Of 14 patients in CTP B class, 2 died 8 and 14 weeks after the end of treatment for reasons not related to antiviral treatment, 1 underwent OLT and is still HCV RNA positive, the remaining 11 showed an improvement in laboratory parameters and in CTP score. Of 11 patients treated for HCC before antiviral therapy, 8 had no evidence of recurrence, 1 in CTP B class underwent OLT and 2 experienced HCC recurrence.

## Discussion

This is a real life study on IFN-free treatment of GT3 patients with advanced fibrosis/cirrhosis. Among the 31 patients (mITT) with severe liver disease who did not achieve SVR main predictors of virological failure across SOF/DCV and SOF/VEL were history of prior Peg-IFN/

RBV failure and presence of baseline NS5A-RASs.

Patients with advanced fibrosis/cirrhosis are the most challenging among HCV infected. In our study they demonstrated 10% increase in response rates from SOF/DCV to SOF/VEL picking to 95.7%. As all our patients had a severe liver damage, the impact of previous Peg-IFN experience was amplified: SVR12 were 20% lower than in naïve. Overall, 70% of relapsers had Peg-IFN/RBV failure history; of 28 SOF/DCV treated who relapsed, 19 were Peg-IFN/RBV failures as all the 3 (mITT) relapsers to SOF/VEL. In this study, baseline NS5A-RASs were observed in 6.4% of cases, 4.9% in naïve and 6.1% in Peg-IFN/RBV experienced patients (p = 0.6). These rates appear lower that the 16% rates reported in other studies [20] but are in line with other findings from Italy [21]. The most common were Y93H, A30K/V and L31F. Of 21 patients with baseline RASs, 66.7% achieved SVR, 100% among SOF/VEL, 33.3% among SOF/DCV (p = 0.001).

Presence of baseline NS5A-RASs was associated with relapse in 33.3% of patients, while 3 additional relapsers (9.6%) developed RASs at the time of virological failure. According with our results and due to the low rate of baseline NS5A-RASs, sequencing might be avoided before using SOF/VEL. Of note, concomitant presence of baseline NS3-RASs, was observed in 16% of patients with NS5A-RASs (data not shown).

The treatment regimen used in patients with GT3 has rapidly changed in the last 2 years [22,23,4,5]. Uptake of SOF/DCV was largely implemented for 12 or 24 weeks depending on the severity of liver disease. In ALLY3+ study, SVR rates higher than 80% were shown with the addition of RBV to SOF/DCV and provided the basis for recommending 12-week duration in patients with cirrhosis, including patients with decompensated disease [4]. RBV is recommended for 24 weeks in cirrhotic experienced patients [12] although no data from large trials are available. Our study based on physician preferences in real life suggests that in cirrhotics SVR12 can be as high as 89.3% without RBV. The corresponding rate in the subgroup of 64 patients who were treated for 24 weeks with RBV was 81.7%. (p = 0.17) No difference by RBV use were also found in cirrhotics experienced (69%% vs 65%, p = 1.0). Therefore, with the limitation of a selection bias determined by the decision to add RBV done on physician’s choices, rather than on randomization, our results suggest that in GT3 and compensated cirrhosis, on treatment with SOF/DCV, RBV and related side effects might be avoided, provided that a course of 24 weeks is adopted. Our results are in keeping with those reported by Hezode et al in GT3 patients with advanced liver disease from the French early access program [24] and with those by Cheung et al on mainly decompensated patients from the UK expanded access program [25].

Of 53 (mTT) cirrhotic patients treated with SOF/VEL, only 32% received RBV. Again physician’s choices appear different from the EASL guidelines recommending SOF/VEL plus RBV for 12 weeks in all Peg-IFN/RBV-experienced [13]. In the ASTRAL-3, patients with cirrhosis and previous treatment history were 37, 33 (89%) achieved SVR [5]. In our real life experience, 23 (mITT) patients who received SOF/VEL were Peg-IFN/RBV-experienced and had cirrhosis; of 14 who were not treated with RBV, only 1 did not achieve SVR.

AASLD recommends RBV in GT3 patients with baseline NS5A-RASs and F3 if undergoing SOF/VEL [14]. Our patients who received SOF/VEL and showed baseline NS5A-RASs were all non-cirrhotic; all achieved SVR without RBV. With the limitation of a small sample size our results suggest that RBV can be avoided in patients with GT3 and compensated cirrhosis and confirm in a real life experience the efficacy of SOF/VEL regimen in GT3.

While further strides on treatment of patients with GT3 with decompensated disease [26] may be required, the patients who were in CPT class B in our study achieved SVR in all but one case. None of them had NS5A-RAS at baseline. Although the number of these patients is expected to decline [27,28], it is desirable not to use RBV in patients with such advanced disease even for only 12 weeks. The exclusion of NS5A-RASs at baseline may be relevant only in this setting. Given that the coming combinations of Glecaprevir and Pibrentasvir [29] and SOF/VEL/VOX [30, 31] containing protease inhibitors are contraindicated in patients with CPT class B and C, SOF/VEL combination will be the only feasible treatment for these patients.

Genetic polymorphisms of PNPLA3GG had not been investigated in previous studies. They did not result predictive of failure (Table 2). However, in agreement with the reported association of this genetic marker with progression of liver disease, numerically higher rates of GG carriers among patients with F4 fibrosis as compared to patients with less severe disease (9.9% vs 5.2%), were observed. These results are in line with recent evidence that support, in patients with decompensated cirrhosis, association between improvement in liver disease outcomes after DAA and lack of PNPLA3GG [32].

We acknowledge that the observational, non-randomized design of this study might represent a limitation and that the study was not designed to compare different treatment strategies. However, strength of the present study is the large number of patients and the excellent SVR12 without RBV. The study includes Italian centers from North and South treating no less than 150 HCV patients per year and representative of the real practice in our Country.

In conclusion, this real world cohort of GT3-infected patients treated with IFN-free regimens confirm efficacy of registration studies. Our results suggest progressive improvements in effectiveness and safety of IFN-free regimens in GT3, confirm previous evidence showing that RBV is not needed in combinations including DCV for 24 weeks or VEL for 12 weeks. Peg-IFN/RBV experience and NS5A-RASs predict relapse. Relevance of RASs seems to decline with the availability of SOF/VEL.

## Supporting information

S1 Fig The complete database of this study is available as attached Excel file. (XLSX)

## Abbreviations

DAA: (Direct acting antivirals)
GT3: Genotype 3
F3: Fibrosis stage 3
F4: Fibrosis stage 4
SOF: Sofosbuvir
DCV: Daclatasvir
VEL: Velpatasvir
NS5ARAS: Resistance associated substitutions in the NS5A region of HCV genome
IFN: Interferon
RBV: Ribavirin
PNPLA: Patatin-like phospholipase gene
IL28B: Interleukin 28 gene
